# A diagnostic approach to mediastinal masses in clinical practice

**DOI:** 10.1093/bjro/tzaf009

**Published:** 2025-05-08

**Authors:** Rebecca Mura, Svitlana Pochepnia, Daria Kifjak, Natallia Khenkina, Helmut Prosch

**Affiliations:** Department of Medicine and Surgery (diMeC), University of Parma, Parma 43125, Italy; Christian Doppler Laboratory for Machine Learning Driven Precision Imaging, Department of Biomedical Imaging and Image-guided Therapy, Medical University of Vienna, Vienna 1090, Austria; Christian Doppler Laboratory for Machine Learning Driven Precision Imaging, Department of Biomedical Imaging and Image-guided Therapy, Medical University of Vienna, Vienna 1090, Austria; Postgraduation School in Radiodiagnostics, University of Milan, Milan 20122, Italy; Christian Doppler Laboratory for Machine Learning Driven Precision Imaging, Department of Biomedical Imaging and Image-guided Therapy, Medical University of Vienna, Vienna 1090, Austria

**Keywords:** mediastinal tumours, CT, MRI

## Abstract

Mediastinal masses represent a heterogeneous group of entities characterized by a variety of histopathological and radiological features. Imaging plays a pivotal role in the detection and interpretation of mediastinal abnormalities. CT remains the modality of choice due to its high spatial and temporal resolution and its ability to assess tissue composition, including the detection of fluid, fat, and calcifications. MRI represents a complementary tool in specific scenarios, such as differentiating complicated cysts from solid lesions or identifying intracellular fat content, as seen in thymic hyperplasia. The differential diagnosis of mediastinal masses relies primarily on the location of the mass and tissue composition, integrated with clinical characteristics of the patient. This review discusses the most common mediastinal masses in adults, providing a practical approach to their differentiation mainly based on the predominant density pattern and location.

## Introduction

Mediastinal masses include a variety of entities with a broad spectrum of histopathological and radiological characteristics. Consequently, a multidisciplinary assessment is recommended to integrate clinical data with imaging findings.^1^ The assessment of anatomical location and tissue characterization of mediastinal lesions plays a pivotal role in enabling the radiologist to provide a differential diagnosis. In this review, we present the most common mediastinal lesions and offer a practical approach for differentiating them based on their predominant density pattern and anatomical location.

## Imaging modalities

The conventional imaging approach to mediastinal masses is based on a stepwise assessment from chest radiograph (CXR) to Computed Tomography (CT), then to positron emission tomography (PET)/CT and/or Magnetic Resonance Imaging (MRI), and diagnostic intervention (ie, biopsy) if needed.^2^^,^^3^ The suspicion for mediastinal lesions is often raised on CXR performed for other purposes but it is of limited value for characterizing these lesions.^4^^,^^5^ The American College of Radiology (ACR) Appropriateness Criteria recommend either contrast-enhanced CT or MRI as the imaging modality of choice for patients with an mediastinal mass, as both allow the evaluation of size, location, margins, density or intensity, and enhancement.^2^ Additionally, multiplanar reconstructions (MPRs) are extremely useful in identifying the origin of mediastinal masses and their spatial relationships with adjacent structures. The main advantages of CT consist of higher spatial and temporal resolution and fast acquisition time, while MRI provides superior tissue characterization and ability to detect invasion across tissue planes.^2^^,^^6^ Thus, except for specific cases (eg, distinguishing complicated cysts from solid lesions or thymic hyperplasia from other thymic masses), the diagnostic performance of CT is comparable to MRI and remains the imaging modality of choice for the initial assessment of a mediastinal lesion.^7–9^ 18-Fluoro-2-deoxy-D-glucose (FDG)-PET/CT offers limited additional value beyond CT, except for primary mediastinal lymphoma staging and treatment response evaluation.^2^^,^^10^ Additionally, 18-F-FDG-PET/CT shows higher standardized uptake values (SUVs) in thymic epithelial tumours (TETs) than in thymic hyperplasia or low-risk thymoma.^2^^,^^11^^,^^12^

## Diagnostic approach

To accurately characterize mediastinal masses and propose a meaningful differential diagnosis, it is crucial to consider the anatomic location, predominant density pattern, and relevant clinical factors.^7^ Age is particularly important, as the prevalence and types of mediastinal abnormalities vary significantly across different age groups.^7^

### Localization of mediastinal abnormalities

**Figure 1.**
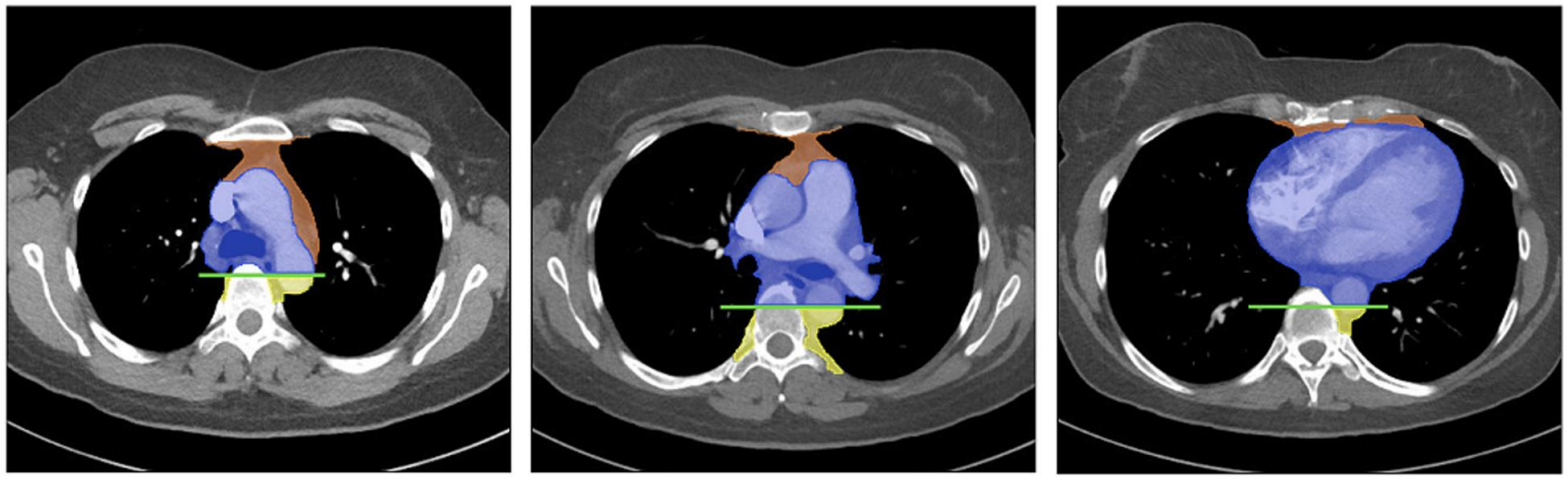
International Thymic Malignancy Interest Group (ITMIG) classification of mediastinal compartments (orange: prevascular; blue: visceral; yellow: paravertebral).

The mediastinum is the central thoracic anatomic compartment situated between the lungs, extending longitudinally from the thoracic inlet to the diaphragm, and containing several vital structures.^4^ Despite the absence of physical boundaries, it is conventionally divided into compartments to facilitate differential diagnoses and surgical treatment plans.^4^ Over the years, several classifications have been proposed and Felson’s CXR-based classification has been widely used over time^13^ but cannot easily applied to cross-sectional imaging. In 2014, the International Thymic Malignancy Interest Group (ITMIG) proposed a classification scheme based on anatomical landmarks easily identified on cross-sectional imaging, particularly on CT.^4^ By this classification system, the mediastinum is divided into three compartments: the prevascular, visceral, and paravertebral compartment.^4^^,^^7^ (Table 1, Figure 1).

**Table 1.** ITMIG CT-based classification of mediastinal compartments (adapted from Carter et al).

Compartment	Boundaries	Major Content
Prevascular	Superior: thoracic inlet Inferior: diaphragm Anterior: sternum Lateral: parietal mediastinal pleura Posterior: anterior aspect of the pericardium	Thymus Fat Lymph nodes
Visceral	Superior: thoracic inlet Inferior: diaphragm Anterior: posterior boundaries of the pre-vascular Posterior: vertical line connecting a point on each thoracic vertebral body 1 cm posterior to its anterior margin	Non-vascular: trachea, oesophagus, lymph nodes Vascular: heart, thoracic aorta (ascending-arch-descending), superior vena cava, intrapericardial pulmonary arteries, thoracic duct
Paravertebral	Superior: thoracic inlet Inferior: diaphragm Anterior: posterior boundaries of the visceral Posterolateral: vertical line against the posterior margin of the chest wall at the lateral margin of the transverse process of the thoracic spine	Thoracic spine Paravertebral soft-tissues

### Density pattern

The main density patterns in mediastinal lesions include soft-tissue, fat, water, calcium, and high contrast enhancement. On CT fat-containing lesions typically show an average attenuation of −40 to −120 Hounsfield Units (HU), while on MRI they appear as areas of high-intensity on T1- and T2-weighted images, with loss of signal on fat-saturated sequences.^6^ CT features of typical cystic lesions include: (a) an oval or tubular mass with a well-defined thin wall; (b) homogeneous water attenuation (0-20 HU); (c) no content enhancement; (d) no evidence of adjacent structures infiltration.^6^^,^^14^ When CT findings are indeterminate, further evaluation with MRI may be recommended to demonstrate high-signal intensity of fluid-containing cysts on T2-weighted images.^14^ Hypervascular mediastinal masses, characterized by intense contrast enhancement, require dynamic imaging for the characterization of specific enhancement patterns.^15^ Finally, soft-tissue components typically measure 40-60 HU on non-contrast CT with variable contrast enhancement, whereas calcifications are characterized by high attenuation (around 1000 HU) and different forms (eg, punctate, coarse, curvilinear).^4^

The following sections describe the most common mediastinal masses in adults, grouped according to their compartment of origin and predominant density pattern, as summarized in Table 2.

**Table 2.** Classification of the most common mediastinal lesions according to their predominant density pattern.

Compartment	Fat	Water	Soft-tissue	Intense contrast-enhancement	Calcification
Prevascular	Mature teratoma	Thymic cyst	Thymic hyperplasia	Hypervascular nodal metastasis	Primary tumour (eg, teratoma, lymphoma)
	Thymolipoma	Pericardial cyst	Thymoma	Paraganglioma	Metastasis from mucinous tumours
	Immature teratoma	Cystic thymoma	Thymic carcinoma	Hemangioma	Thyroid goitre
	Teratocarcinoma	Cystic teratoma	Thymic carcinoid	Ectopic parathyroid adenoma	Granulomatous processes or pneumoconiosis
	Lipoma	Lymphangioma	Lymphoma	Arteriovenous malformation	
	Liposarcoma	Cystic tumour degeneration	Thyroid goitre		
	Fat necrosis	Abscess	SMARCA-4 deficient tumours		
	Lipomatosis		Castleman disease		
	Congenital hernia (morgagni hernia)				
Visceral		Bronchogenic cysts	Oesophagus neoplasms	Hypervascular nodal metastasis	Metastasis from mucinous tumours
		Oesophageal duplication cysts	Hiatal hernia	Arteriovenous malformation	Granulomatous processes or pneumoconiosis
				Paraganglioma	
Paravertebral	Lipoma	Intrathoracic meningocele	Neurogenic tumours (eg, schwannoma, neurofibroma)	Hypervascular nodal metastasis	Metastasis from mucinous tumours
	Liposarcoma	Neuroenteric cyst	Extra-medullary haematopoiesis	Paraganglioma	
	Lipomatosis	Mullerian cyst	Infectious spondylodiscitis	Hemangioma	
	Congenital hernia (bochdalek hernia)	Cystic schwannoma		Arteriovenous malformation	

## Prevascular compartment

### Fat-containing lesions

#### Teratoma

Teratomas are the most common mediastinal germ-cell neoplasms (GCNs). They mostly occur during the second to fourth decades and contain more than one of the primitive germ-cell layers (ectoderm, mesoderm, endoderm).^16^^,^^17^ The vast majority of teratomas are *mature teratomas*, hence histologically well-differentiated and benign.^16^ Rarely, they may contain undifferentiated neuroectodermal tissue (*immature teratoma*), which is considered potentially malignant, or components of carcinoma/sarcoma (*teratocarcinoma*), regarded as malignant.^16^

**Figure 2.**
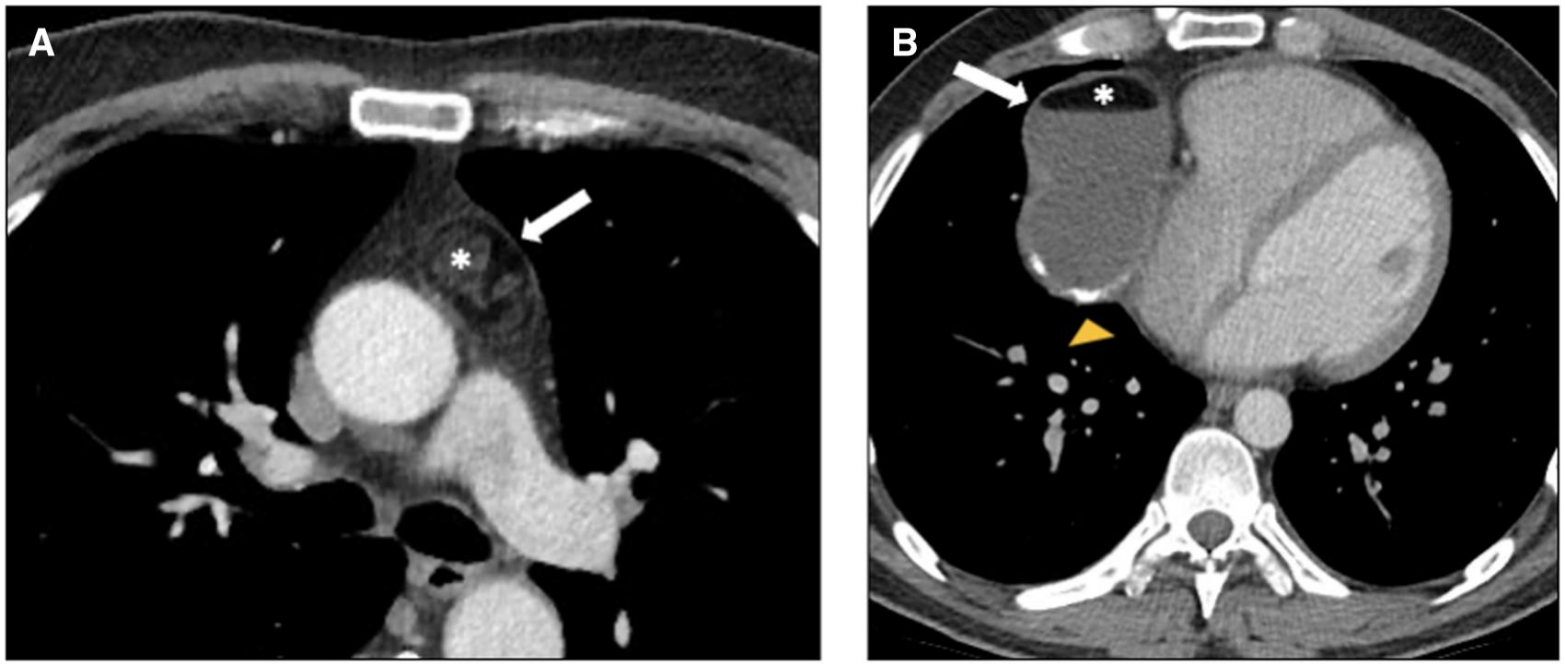
Mature teratoma. (A) Contrast-enhanced axial CT image shows a heterogeneous mass in the left prevascular mediastinum, characterized by encapsulated fatty tissue (arrow) intermixed with soft-tissue components (asterisk). (B) Contrast-enhanced axial CT image shows a right prevascular mediastinal mass with well-defined margins, heterogeneous density, containing both fluid and fat (asterisk) with the characteristic fat-fluid level (solid arrow) and rim-like calcifications (yellow arrowhead).

On cross-sectional imaging, teratomas exhibit well-defined, rounded, or lobular margins and a heterogeneous appearance.^5^^,^^16^^,^^17^ Fat is present in 75% of lesions, often with characteristic fat-fluid levels, and fat-suppressed MR sequences can be helpful to confirm this content.^5^^,^^16^^,^^17^ Rim-like or tooth-like calcifications are commonly observed^16^ (Figure 2). Some mature teratomas can exhibit a predominantly cystic morphology with internal septations, referred to as *cystic teratomas*.^16^ Differentiating between mature and immature teratomas is challenging on imaging as the immature elements can be represented only by small solid-tissue components. A combination of fluid, soft-tissue, calcium, and fat attenuations within a well-defined mass is highly specific for a mature teratoma. In contrast, a predominantly solid, enhancing lesion with necrotic or haemorrhagic areas raises suspicion for teratocarcinoma.^16^

#### Thymolipoma

**Figure 3.**
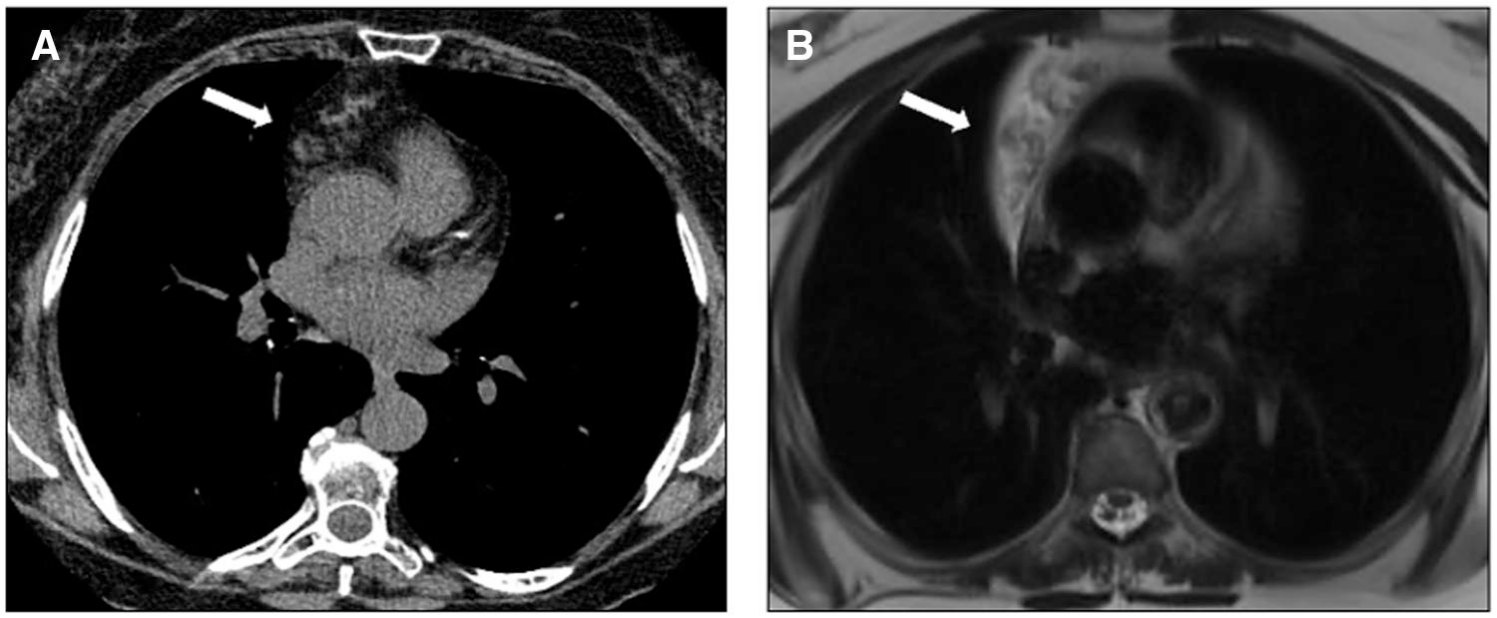
Thymolipoma. (A) Non-contrast axial CT image shows a fatty mass in the right prevascular compartment intermixed with linear whorls of soft-tissue (arrow), corresponding to adipose and thymic tissue, respectively. (B) Axial T2-weighted MR image of the chest shows the prevascular mass (arrow), characterized by predominant high-signal intensity due to the fat component and intermediate signal intensity from the thymic tissue.

*Thymolipomas* are benign, slow-growing lesions, composed of a mixture of adipose and thymic tissue in variable proportions, with the fat content constituting up to 50%-85% of the lesion.^18^^,^^19^ They typically occur in young adults and are often associated with myasthenia gravis or thyroid disorders.^18^ On CT, thymolipomas appear as encapsulated fatty lesions with linear whorls of soft-tissue thymic component.^5^^,^^17^ Similarly, on MRI, these lesions demonstrate high-signal intensity on T1- and T2-weighted images due to the fat content, with signal suppression on fat-suppressed sequences, alongside intermediate intensity from the thymic tissue^5^^,^^17^ (Figure 3).

#### Lipoma and liposarcoma

Lipomatous neoplasms are mesenchymal tumours that can occur in both prevascular and paravertebral compartments.

*Lipomas* are benign lesions made up of mature adipocytes. They account for approximately 2% of mediastinal neoplasms, with a low risk of malignant degeneration.^5^ On CT, they appear as encapsulated, homogeneous, fat-attenuated lesions without contrast enhancement.^5^ Conversely, the presence of soft-tissue components, fibrous bands, or internal septa with contrast enhancement are features consistent with a diagnosis of *liposarcoma.*^5^^,^^20^

*Liposarcomas* are rare primary malignant mediastinal tumours (accounting for 0.1%-0.75% of all mediastinal lesions), typically found in individuals in the fifth to sixth decades of life.^5^^,^^21^ Depending on their grade of differentiation, liposarcomas can demonstrate a variable appearance ranging from fatty lesions nearly indistinguishable from lipomas, to heterogeneous enhancing masses.^20^ On 18F-FDG-PET, liposarcomas usually exhibit higher FDG uptake compared to lipomas.^20^

Differential diagnoses for lipomatous tumours include *mediastinal lipomatosis*, *mediastinal fat necrosis*, and *omental hernia*. *Mediastinal lipomatosis* is a benign condition of excessive fat deposition often linked to steroid use, obesity, or Cushing syndrome.^5^  *Mediastinal fat necrosis* is an inflammatory self-limiting condition, causing acute chest pain, usually after trauma or surgery.^5^ On CT, it appears as a juxta-pericardial fat-attenuation area with surrounding soft-tissue stranding and thickening of the adjacent pericardium.^22^ On 18-F-FDG-PET/CT, fat necrosis could show uptake of FDG, which is expected to resolve on follow-up.^6^ Finally, o*mental hernia* refers to herniation of omental fat into the mediastinum, either due to congenital (ie, Morgagni and Bochdalek hernias for prevascular and paravertebral compartment, respectively) or acquired defects (trauma or postoperative complications).^5^^,^^17^^,^^20^ MPRs help identify diaphragm discontinuity, which allows to differentiate hernias from lipomatous tumours.^5^^,^^20^

### Cystic lesions

#### Thymic cyst

A purely cystic mass with no soft-tissue components or septa in the thymic bed can be reliably classified as a *thymic cyst*.^4^ Thymic cysts account for 1%-3% of all mediastinal lesions and can be either congenital or acquired, arising after radiation therapy (RT), thoracotomy, or in relation to autoimmune conditions (eg, Sjogren syndrome).^5^

**Figure 4.**
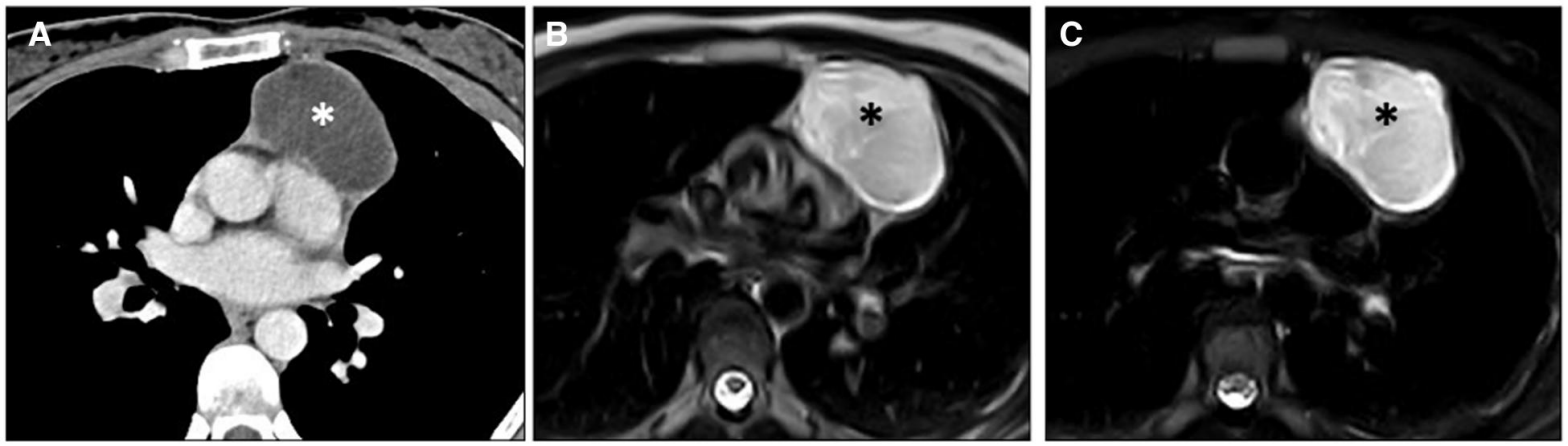
Thymic cyst. (A) Contrast-enhanced axial CT image of the chest shows a prevascular uniloculated thin-walled lesion (white asterisk) with fluid attenuation, in the typical location of the thymic structure. (B) Axial T2-weighted MR image shows hyperintense signal intensity of the lesion (black asterisk), maintained in fat-suppressed sequence (C) and confirming its water content.

Congenital cysts are typically unilocular, thin-walled lesions filled with clear fluid, whereas acquired cysts are more often multilocular and may have variable wall thickness.^5^ Distinguishing between these types is important as acquired cysts may be associated with malignancies and tend to recur after resection. When haemorrhage or infection occurs, the cysts exhibit higher CT attenuation, potentially leading to a misdiagnosis as a solid mass. In such cases, further characterization with MR imaging should be performed^5^^,^^16^ (Figure 4).

#### Lymphangioma

*Lymphangioma* is a rare congenital vascular malformation resulting from an embryologic failure of lymphatic drainage into the venous system.^23^ It is typically found in the cervical and axillary regions, occasionally extending into the mediastinum.^23^ Less than 1% of cases are confined to the chest, more often in the prevascular mediastinum.^23^ Although lymphangiomas are generally diagnosed in childhood, a few adult cases have been reported, possibly due to slow-growing congenital forms or secondary to chronic lymphatic obstruction (eg, following RT, chronic infection, or surgery).^23^^,^^24^ Lymphangiomas are classified based on the size of their dilated lymphatic channels into simple, cavernous, or cystic forms (ie, *cystic hygromas*) and can be either unilocular or multilocular, with cysts of variable size.^5^

On CT, lymphangiomas appear as lobulated lesions with fluid attenuation, which envelop rather than displace adjacent structures.^5^^,^^14^ Calcification and contrast enhancement are uncommon.^5^^,^^14^ If CT findings are ambiguous, MRI can be used to confirm the fluid content; however, on T1-weighted images, the lesions can exhibit heterogeneous signal intensity, depending on their content.^5^^,^^14^ A multilocular cystic lesion with septations or soft-tissue components in the prevascular mediastinum should also be differentiated from *cystic teratoma* and *cystic thymoma*.^4^

#### Pericardial cyst

**Figure 5.**
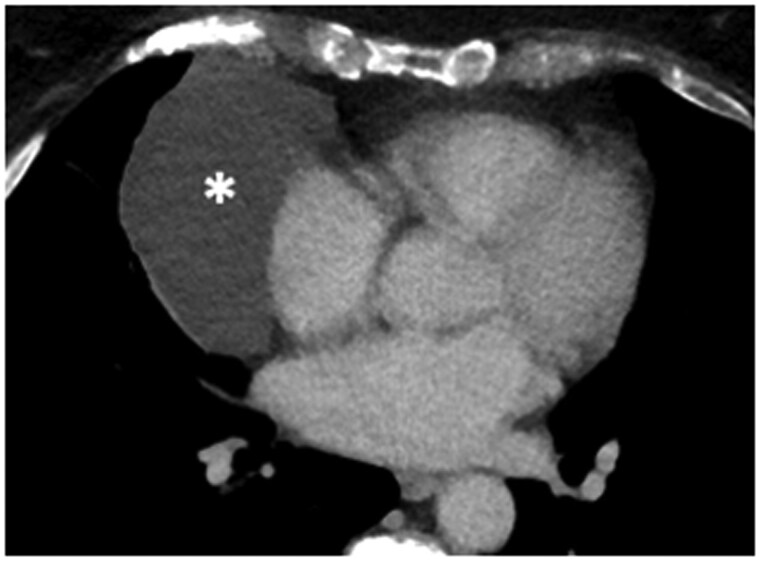
Pericardial cyst. Contrast-enhanced axial CT image of the chest reveals a lesion (asterisk) in the right cardiophrenic angle, characterized by fluid density and direct contiguity with the pericardial surface.

A well-defined unilocular lesion of fluid attenuation with imperceptible walls in the cardiophrenic angle is highly suggestive of a *pericardial cyst*.^6^ Pericardial cysts are benign congenital anomalies resulting from abnormal formation of coelomic (somatic) cavities.^6^^,^^25^ They can develop anywhere along the pericardial lining, but most commonly occur in the anterior cardiophrenic angles, particularly on the right side.^6^^,^^25^ Pericardial cysts exhibit the typical imaging features of cystic lesions on both CT and MRI^16^^,^^26^ (Figure 5).

### Soft-tissue lesions

#### Thymic hyperplasia

*Thymic hyperplasia* typically manifests in younger adults as uniform thymic enlargement, while in patients over 40 years, it may appear as a soft-tissue in the thymic bed resembling normal thymus.^4^ Histologically, it is classified into 2 subtypes: true (rebound) and lymphoid (follicular) hyperplasia. True hyperplasia is characterized by more than a 50% increase in thymic volume with normal histology, often triggered by stressors such as chemotherapy, RT, or corticosteroids.^4^^,^^5^^,^^16^ Lymphoid hyperplasia, on the other hand, is distinguished by the presence of lymphoid follicles, with or without glandular enlargement, and is commonly associated with immunologic disorders like myasthenia gravis or collagen vascular disease.^4^^,^^5^^,^^16^ On CT, true hyperplasia presents as diffuse symmetric enlargement, whereas lymphoid hyperplasia may appear as a normal thymus, diffuse enlargement, or occasionally as a focal mass.^4^

**Figure 6.**
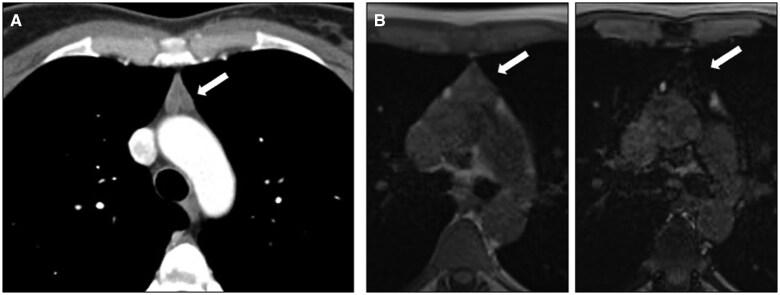
Thymic hyperplasia. (A) Contrast-enhanced axial CT shows a diffuse enlargement of the thymus (arrow). (B) Chemical-shift MR imaging demonstrates in-phase homogeneous intermediate signal intensity of the thymus (left) and signal drop on out-of-phase imaging (right), consistent with thymic hyperplasia.

In this setting, in-phase and opposed-phase gradient-echo MRI sequences can be used to detect microscopic fat, which is typical for thymic hyperplasia and normal thymus, and is absent in TETs^4^^,^^7^^,^^27^ (Figure 6).

#### Thymic epithelial tumours

**Figure 7.**
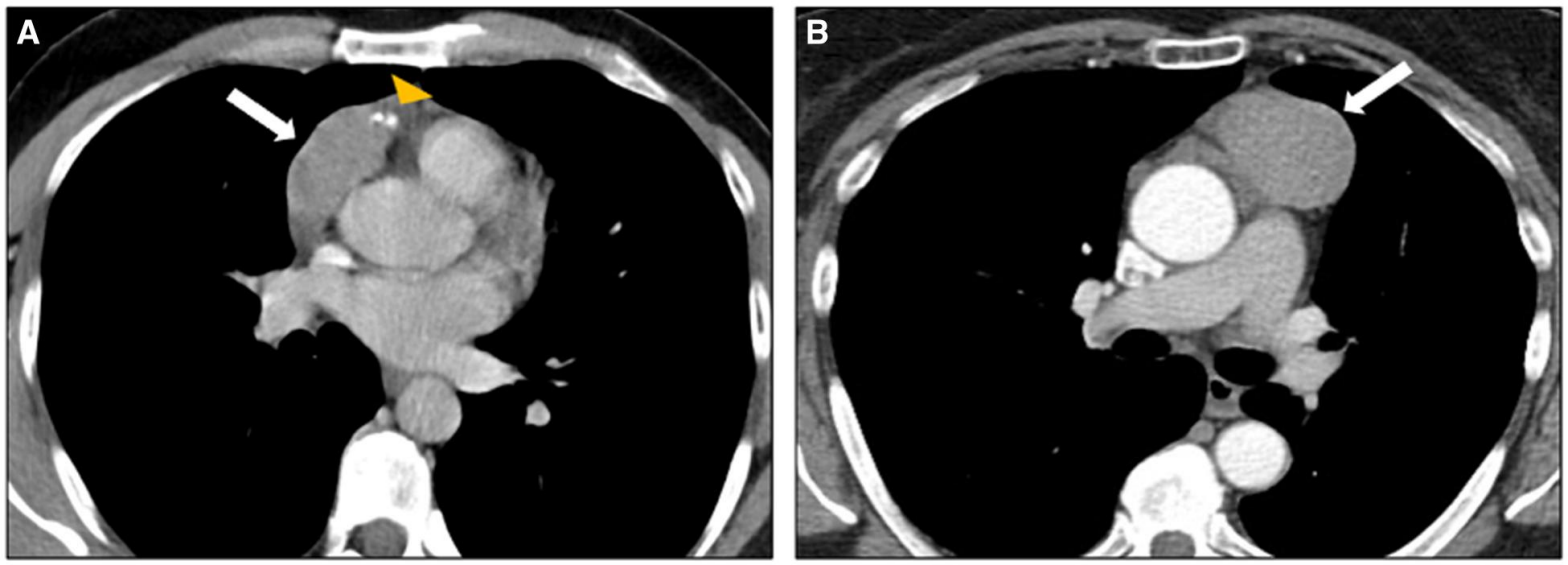
Thymoma. (A, B) Contrast-enhanced axial CT images show soft-tissue enhancing lesions (arrow) of the prevascular mediastinum, with punctate peripheral calcifications (A, yellow arrowhead). Biopsy revealed in both cases a thymoma (B2-B3, stage I Masaoka-Koga).

TETs are the most frequent prevascular solid tumours and include thymomas, thymic carcinomas, and thymic carcinoids.^28^  *Thymomas* account for approximately 20% of these lesions in adults,^29^ with a peak of incidence between 55 and 65 years, and are frequently associated with autoimmune disorders, especially myasthenia gravis.^30^ On CT, thymomas typically present as spherical or ovoid, encapsulated, off-midline soft-tissue lesions with homogeneous attenuation and contrast enhancement (Figure 7). Heterogeneity within the lesion may be due to necrosis, haemorrhage, or cystic change (*cystic thymoma*).^29^^,^^31^ Calcifications in thymoma exhibit various patterns, including curvilinear (along the capsule or fibrous septa), punctate, or coarse.^29^ In advanced stages, pleural or pericardial dissemination (drop metastases) may occur.^4^^,^^5^

Although MRI may be more effective in assessing local invasion, it is not routinely used due to the lack of distinctive features and superior spatial resolution of CT.^29^^,^^31^ On imaging, a reliable differentiation among TET is not feasible.^32^ However, a diagnosis of thymic carcinoma or thymic carcinoid should be considered when a lesion shows aggressive features like increased heterogeneity, irregular margins, local invasion, lymphadenopathy, and pleural or pericardial effusion.^4^^,^^16^^,^^29^ On 18F-FDG-PET/CT, thymic carcinomas and carcinoids typically demonstrate greater FDG uptake than thymomas.^33^^,^^34^ In the diagnostic evaluation of carcinoids, the nuclear medicine technique targeting somatostatin receptor (SSTR) expression has been recently recognized as a valuable tool.^28^ Thymic malignancies are staged using CT according to the ninth edition TNM staging system.^35^ However, the Masaoka-Koga staging system, which strongly correlates prognosis with clinical stage, remains widely used for thymomas.^36^^,^^37^

#### Lymphoma

*Lymphomas* account for up to 20% of mediastinal masses.^16^ Primary mediastinal lymphomas are rare, comprising 1% of all lymphomas^16^ with Hodgkin lymphoma and primary mediastinal large B-cell lymphoma as the main histologic subtypes.^5^^,^^16^ On contrast-enhanced CT, they typically appear as lobulated soft-tissue masses with mild-to-moderate enhancement, although cystic changes and necrosis may be present.^5^ Calcifications are uncommon prior to treatment.^30^ Differentiating lymphoma from other prevascular masses can be challenging; however, unlike other malignancies, lymphomas tend to encase adjacent structures without direct infiltration.^4^^,^^32^ Young age of patients—second to third decades—also supports the diagnosis.^4^^,^^5^ 18-F-FDG-PET/CT remains the preferred imaging modality for lymphoma staging and monitoring disease activity.^38–40^ However, whole-body (WB)-MRI has emerged as viable, radiation-free alternative particularly for younger patients.^41^^,^^42^ Differential diagnosis, particularly in young patients (third to fourth decades), includes *Castleman disease*, a non-clonal lymphoproliferative disorder. Its most common form is the unicentric hyaline vascular type, which typically presents as a non-invasive mass (in 50% of cases), but may also appear as an infiltrative mass with lymphadenopathy or as matted lymph nodes without a discrete mass.^43^

Another rare differential diagnosis is *SMARCA4-deficient thoracic sarcomas* (SMARCA4-DTS), a recently described undifferentiated sarcoma that most commonly occur in the mediastinum of young-middle aged male smokers.^44^^,^^45^ These tumours present as ill-defined masses with heterogeneous enhancement, frequently crossing anatomical compartments and demonstrating strong avidity on 18-F-FDG-PET/CT.^45^^,^^46^ At diagnosis, confluent, necrotic, ill-defined lymphadenopathies with surrounding infiltrate are observed, highlighting the lymphatic spread of these tumours, which is uncommon in other types of sarcomas, and further complicating differentiation from lymphomas.^46^^,^^47^ Thus, diagnosis requires histopathological and immunohistochemical confirmation.

#### Thyroid goiter

An encapsulated, lobulated, heterogeneous mass that is intrinsically hyperdense (70-85 HU) and continuous with the cervical thyroid gland can be reliably diagnosed as *mediastinal goitre*.^4^ Intrathoracic goitre accounts for 3%-6% of mediastinal masses, occurring more commonly in adults. 1%-2% can be ectopic without any connection to the cervical thyroid.^4^^,^^5^^,^^16^ On contrast-enhanced CT, these lesions typically exhibit homogeneous, sustained enhancement, although cystic changes and calcifications are common.^5^^,^^16^ Thyroid goitres can extend into the visceral compartment and cause tracheal displacement or compression.^4^ Loss of fascial planes or the presence of adjacent lymphadenopathies should raise suspicion for thyroid malignancy.^7^

## Visceral compartment

### Cystic lesions

#### Foregut duplication cysts

**Figure 8.**
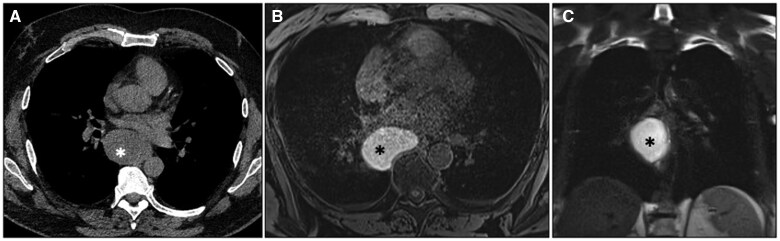
Bronchogenic cyst. (A) Non-contrast CT axial image shows a homogeneous lesion (asterisk) in the right visceral mediastinum under the level of the carina, with attenuation values slightly higher than fluid. (B, C) Axial T1-weighted and coronal T2-weighted MR images show high-signal intensity, consistent with a cystic lesion with a heterogeneous content.

*Foregut duplication cysts* are congenital anomalies arising from abnormal budding of the embryonic foregut and tracheobronchial tree. These lesions include bronchogenic and oesophageal duplication cysts.^48^^,^^49^  *Bronchogenic cysts* account for 50%-60% of mediastinal cystic lesions and are most commonly located in the visceral compartment (less frequently in the paravertebral mediastinum) near the tracheal carina or, less commonly, in the paratracheal or hilar region, often right-sided.^49^^,^^50^ Although most cases are detected during the neonatal period, incidental findings in adults are also possible.^51^ These cysts exhibit variable attenuation on CT—due to their mixed content of fluid and proteinaceous mucoid material—and heterogeneous signal intensity on T1-weighted MR images^49^^,^^50^ (Figure 8). Air within the cyst is uncommon and suggests infection.^14^^,^^49^^,^^50^

*Oesophageal duplication cysts* are more commonly detected in children, within or adjacent to the oesophageal wall. A ^99m^Technetium-pertechnetate scan can help identify oesophageal cysts containing ectopic gastric mucosa (present in up to one-third of cases), which are at higher risk for complications such as perforation, infection, or haemorrhage. CT and MRI findings closely resemble those of bronchogenic cysts, though oesophageal cysts typically have thicker walls and are generally closer to the oesophagus.^6^^,^^14^^,^^52^

### Soft-tissue lesions

#### Oesophagus neoplasms

Up to 80% of o*esophagus neoplasms* are malignant, primarily squamous cell carcinoma and adenocarcinoma, both of which are more common in the seventh decade of life.^53^^,^^54^ These tumour types are indistinguishable on imaging, but most adenocarcinomas involve the distal oesophagus and the stomach. Although CT is not the primary imaging modality for the oesophagus, it can demonstrate focal asymmetric thickening (>5 mm) or a soft-tissue mass in the oesophageal wall, which may progress to circumferential involvement and invasion of adjacent structures.^53^^,^^54^ In contrast, mesenchymal neoplasms, such as leiomyomas and gastrointestinal stromal tumours (GISTs), typically exhibit well-defined smooth margins without peritumoral invasion or lymphadenopathies; however, differentiating between them is crucial due to the malignant potential of GISTs.^53^^,^^54^ Differential diagnoses for oesophageal wall thickening also include inflammatory conditions, such as gastroesophageal reflux disease, associated with *hiatal hernia*.^55^

## Paravertebral compartment

### Cystic lesions

#### Intrathoracic meningocele

*Intrathoracic meningocele* is an anomalous saccular protrusion of the leptomeninges through an intervertebral foramen or bone defects containing cerebrospinal fluid.^14^^,^^20^ More common in adults, it can be associated with neurofibromatosis type 1.^20^ Meningoceles typically occur in the thoracic spine, especially between T3 and T7, and are usually right-sided, probably due to the aorta’s position on the left. CT scans reveal sharply defined, homogeneous, low-attenuation lesions, with adjacent vertebral abnormalities or enlarged foramina.^4^^,^^14^^,^^20^ However, MRI is the gold standard for differentiating meningocele from other paravertebral lesions (eg, neurogenic tumours), as it confirms their cystic nature and connection to the thecal sac.^4^^,^^14^^,^^20^

Extramedullary *cystic schwannomas* should be considered in the differential diagnosis, as they may present cystic components or cystic degeneration, appearing as areas of high-signal intensity on T2-weighted sequences with peripheral enhancement on T1-weighted images.^14^^,^^56^ Differential diagnoses should also include *neuroenteric* and *Müllerian cysts*, both of which display similar imaging features to intrathoracic meningocele. *Neurenteric cysts* are a rare type of foregut cysts resulting from abnormal portioning of the embryonic notochordal plate.^57^ They are typically intradural and extramedullary, located ventral to the spinal cord, most commonly in the cervical spine.^57^ These cysts are frequently diagnosed during childhood or in the second to third decades, due to respiratory symptoms or associated radiculopathy.^20^^,^^57^ Lastly, *Müllerian* cysts—a recently described benign entity^33^ occurring only in females—can mimic a cystic neural lesion and require pathologic specimens and immunohistochemical staining for diagnosis.^1^^,^^58^

### Soft-tissue lesions

#### Neurogenic tumours

*Neurogenic tumours* are the most common paraspinal masses. The malignancy rate is higher in infants and young children, while in young adults (aged <40 years), nerve sheath tumours are the most common subtype, including benign schwannomas, neurofibromas, and malignant peripheral nerve sheath tumours (MPNSTs).^20^^,^^59–61^ These tumours typically originate from spinal nerve roots or intercostal nerve^59^ and often assume a characteristic “dumbbell” shape when extending through intervertebral foramina.^20^ Benign lesions usually appear as well-defined, fusiform, or spherical soft-tissue lesions spanning several rib interspaces, while larger lesions may become eccentric to the nerve, lobulated, and more heterogeneous.^20^^,^^59^

**Figure 9.**
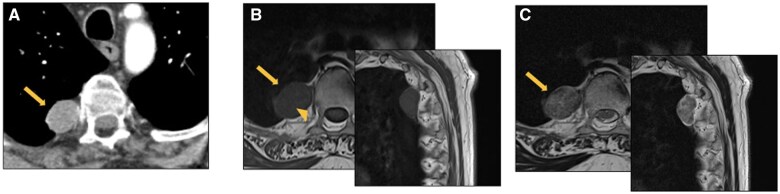
Schwannoma. (A) Contrast-enhanced CT axial image (arterial phase) shows a well-defined rounded soft-tissue lesion (arrow) in the right paravertebral mediastinum. This mass abuts the adjacent spinal nerve (arrowhead), appears hypointense on T1-weighted MR images (B) and moderately hyperintense with central hypointense foci on T2-weighted images (C), representing thickened fascicles (fascicular sign).

CT may reveal the “*split fat sign*” indicating a non-infiltrating mass by showing displaced but intact fat surrounding the neuromuscular bundle. Peripheral calcifications can be seen in about 10% of schwannomas, while adjacent osseous erosion is more common in neurofibromas.^59^ Both schwannomas and neurofibromas show low- to intermediate signal intensity on T1-weighted images. On T2-weighted sequences, neurofibromas may display the “*target sign*”, characterized by a central low-signal zone surrounded by higher intensity.^20^^,^^59^ Schwannomas may show hypointense small foci with a ring-like shape in a more hyperintense mass (“*fascicular sign*”).^20^^,^^26^^,^^59^ MRI is also valuable for assessing intraspinal extension and neural invasion^20^^,^^59^ (Figure 9).

Rapid growth, necrosis, and local invasion are suggestive of malignancy^20^^,^^59^ and 18F-FDG/PET helps in distinguishing malignant tumours from benign neurofibromas.^62^ Additionally, Wasa et al reported high suspicion of MPNSTs in nerve sheath tumours displaying 2 or more of the following MRI features: size >5 cm, peripheral enhancement, perilesional oedema, or intratumoral cystic change.^63^

#### Infectious diseases

Infectious spondylodiscitis usually originates from pyogenic or tuberculous (Pott’s disease) infection via haematogenous spread. It can extend into pre- and paravertebral soft-tissues, affecting single or multiple spinal segments.^20^^,^^64^ Osteolytic destruction may cause anterior vertebral collapse, resulting in the characteristic gibbus deformity in Pott’s disease, while paravertebral abscess forms a “horseshoe” mass around the affected vertebra.^20^^,^^64^^,^^65^ CT and MRI are highly sensitive for detecting early osteolytic changes, with contrast media enhancing paraspinal abscess and disc involvement. On MRI, spondylodiscitis typically shows hypo- to isointense T1-signals and hyperintense T2-signals in the subchondral end plates and intervertebral discs.^20^^,^^64^^,^^65^

#### Extramedullary haematopoiesis

**Figure 10.**
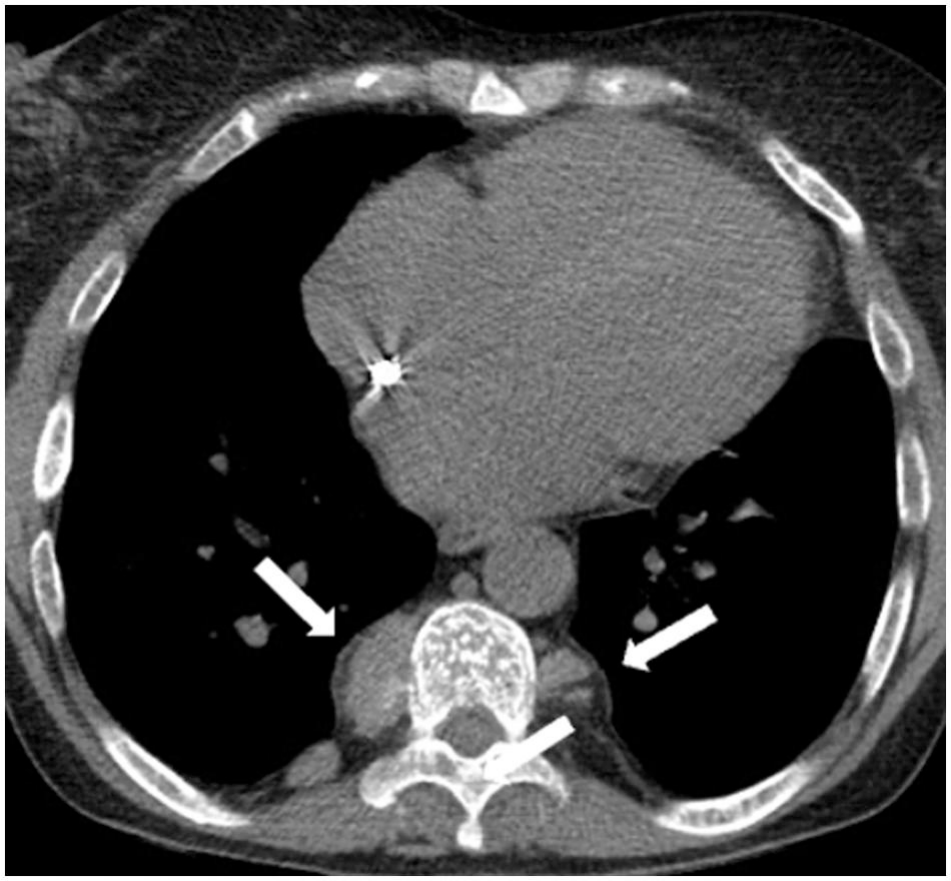
Extramedullary haematopoiesis. Non-contrast axial CT image shows bilateral spinal soft-tissue (arrows) in a patient with beta-thalassemia.

*Extramedullary haematopoiesis* occurs in response to insufficient bone marrow function (eg, myelofibrosis, leukaemia, haemolytic anemias) with the expansion of haematopoietic tissue outside the bone marrow and formation of paravertebral soft-tissue masses, usually in the lower thoracic spine.^66^ These masses are typically bilateral, well-defined, with mild contrast enhancement.^20^^,^^67^ The appearance on CT and MRI varies with the lesion’s haematologic activity. Active lesions show soft-tissue density on CT and intermediate T1- and T2-signals on MRI, while inactive lesions, containing fat and/or iron deposits, exhibit variable CT attenuation and MR signal intensity based on the proportion of these components^68^ (Figure 10).

## Any compartments

### Cystic-like lesions

*Cystic degeneration* can occur in a variety of tumours, including thymomas, Hodgkin lymphoma, GCNs, metastatic lymph nodes, and neurogenic tumours.^14^ It is more likely after RT or chemotherapy but may also present before treatment. Extensive degeneration results in mixed solid and cystic components on CT and MRI and can make it difficult to differentiate between tumour types and other mediastinal cysts.^14^

*Mediastinal abscesses* should also be considered in patients with low-attenuation masses on CT following recent surgery, oesophageal perforation, or infections (eg, retropharyngeal abscess, osteomyelitis, empyema).^5^^,^^14^ Air bubbles and a thick rim-enhancing wall may be present.^14^

### (Intense) Enhancing lesions

#### Hypervascular nodal metastases

*Hypervascular nodal metastases* can originate from intra- or extrathoracic tumours, such as renal cell carcinoma, melanoma, neuroendocrine tumours, and thyroid carcinoma (with the first two being the most prevalent).^15^ PET/CT is often recommended for identifying the primary tumour and staging the disease.^15^

#### Mediastinal hemangioma

Mediastinal haemangioma is a rare benign vascular tumour, accounting for less than 0.5% of mediastinal masses.^15^ It can affect patients of all ages, with a higher incidence in younger individuals (mean age of 35 years).^69^ Histologically, haemangiomas consist of vascular spaces interspersed with various stromal tissues (eg, fat, myxoid or fibrous tissue), leading to a heterogeneous appearance on both CT and MRI. Dynamic imaging reveals gradually increasing enhancement with a centripetal fill-in pattern and persistent delayed enhancement.^6^^,^^15^^,^^69^ Thrombus formation within the vessels is frequent and may calcify as phlebolith, which is a helpful diagnostic feature on CT,^6^^,^^70^ along with the presence of aberrant draining veins.^69^

#### Paragangliomas

Paragangliomas are rare neuroendocrine tumours arising from extra-adrenal chromaffin cells in the parasympathetic or sympathetic ganglia. Mediastinal paraganglia are often located in the prevascular compartment along the great vessels (especially in the aortic-pulmonary window) or in the paravertebral compartment along the sympathetic chain.^20^ Aortopulmonary paragangliomas often occur in patients older than 40 years, while aortosympathetic ones typically affect younger individuals (second to third decades).^15^

**Figure 11.**
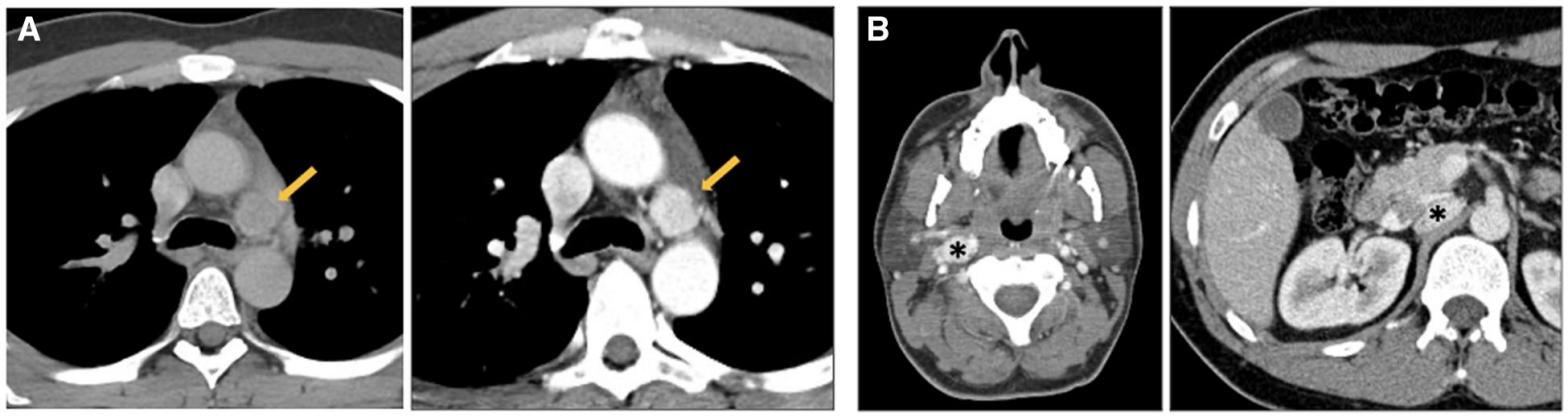
Paraganglioma. (A) Plain (left) and contrast-enhanced axial CT (right) shows a well-defined hypodense lesion (arrow) in the visceral mediastinum (aortopulmonary window) with intense contrast enhancement. (B) Multiple paragangliomas have been identified in other sites of the same patient: at the division of the right common carotid artery (left image) and retrocaval (right image).

On dynamic imaging, paragangliomas show early intense enhancement after contrast administration (Figure 11) but they may also present calcifications or areas of cystic degeneration. MRI reveals a “salt and pepper” pattern, with hyperintense regions (*salt*) representing haemorrhage (on T1- and T2-weighted images) or slow flow within vessels (on T2-weighted images), while the focal signal voids (*pepper*) correlate with the high-velocity flow in other vessels.^6^^,^^15^ PET/CT with 68-Ga DOTATATE is increasingly used for the diagnosis, staging, and follow-up.^71^

#### Ectopic parathyroid adenoma

*Parathyroid adenoma*s are benign, functioning tumours typically found in the neck, but they can be ectopically located in approximately 10% of cases, most often near or within the thymus.^16^ They can occur at any age, but more commonly between fifth and seventh decades.^72^ On both CT and MRI, parathyroid adenomas appear as rounded lesions with smooth margins, early intense enhancement in the arterial phase, and a rapid washout in the delayed phase, allowing for differentiation from lymph nodes and thyroid tissue.^6^ A suspicion can be confirmed by single photon emission CT (SPECT) using ^99m^Tc-sestamibi.^15^

#### Arteriovenous malformation

*Arteriovenous malformations* (AVMs) are high-flow vascular anomalies. On dynamic CT and MRI, they present dilated feeding arteries and draining veins, with or without a central nidus, and no significant soft-tissue components.^15^^,^^73^ After contrast administration, early vein opacification is observed, consistent with shunting.^15^^,^^73^

### Calcified lesions

Calcifications in mediastinal masses can be observed across all compartments and may be associated with both malignant and benign conditions.^7^

**Figure 12.**
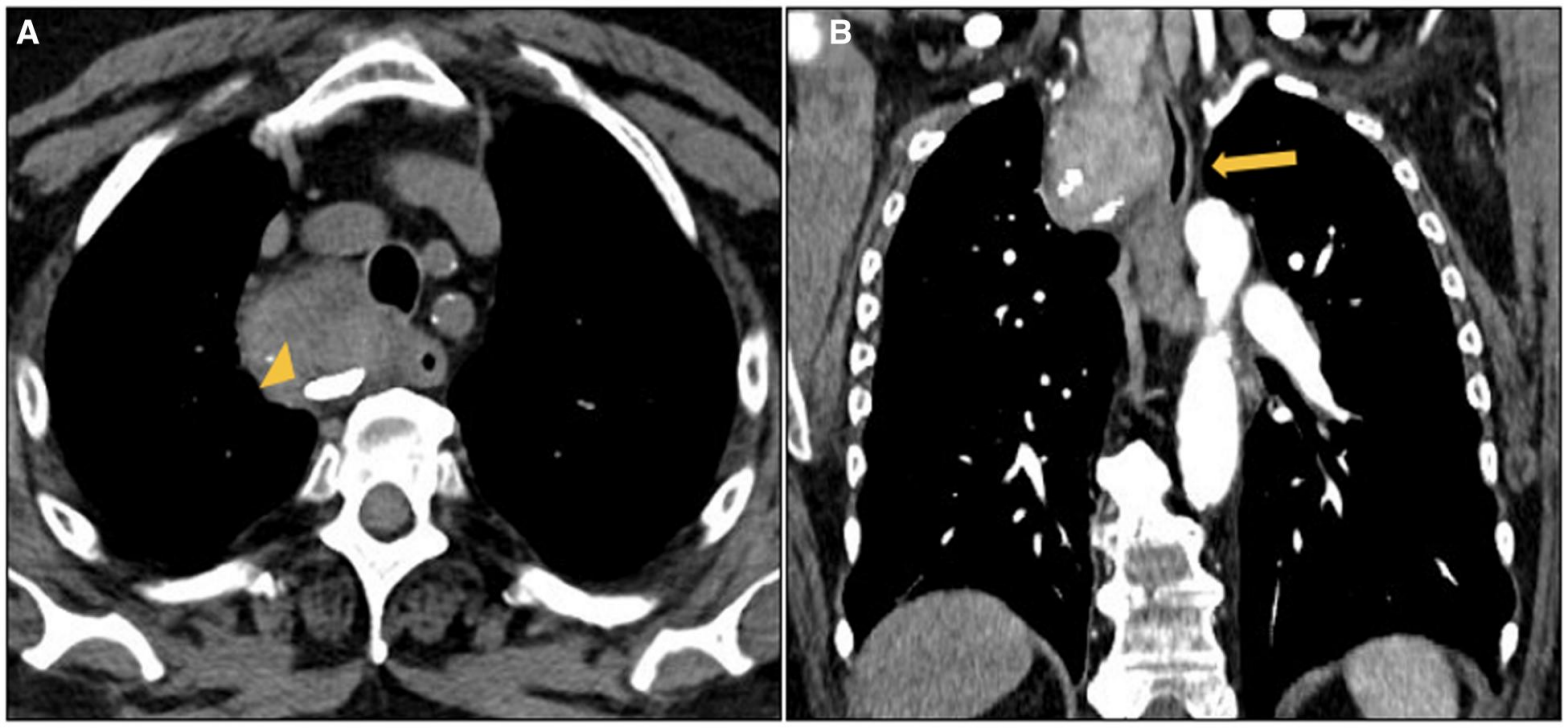
Thyroid goitre. (A) Non-contrast axial CT shows a large, lobulated mass in the visceral mediastinum with cystic changes and peripheral calcifications (arrowhead). (B) Coronal contrast-enhanced CT reconstruction shows the continuity with the right lobe of the cervical thyroid and the compression of the tracheal lumen (arrow).

Primary tumours, such as *teratomas*, often exhibit rim-like or tooth-like calcifications.^5^^,^^74^ M*etastatic mediastinal lymph nodes* from mucinous tumours (eg, ovarian, gastrointestinal cancers) and bone-forming sarcoma may present with calcific deposits^8^ as well as treated malignancies, in particular lymphomas.^5^ Benign conditions can also lead to calcifications, as seen in up to 75% of *thyroid goiters*^5^ (Figure 12).

Finally, calcified lymph nodes may indicate *granulomatous processes* (eg, sarcoidosis, tuberculosis) or *pneumoconiosis* (eg, silicosis). Calcification patterns can vary, including complete nodal calcifications in tuberculosis and “*egg-shell*” or “*icing sugar*” patterns in sarcoidosis and silicosis.^5^^,^^75^^,^^76^

## Conclusion

A wide spectrum of conditions can occur in the mediastinum, making a standardized approach challenging. In this review, we highlight the importance of imaging findings—particularly tissue characteristics and localization within mediastinal compartments—as valuable tools for radiologists in formulating meaningful differential diagnoses.
